# Post-traumatic trigeminal neuropathy. A study of 63 cases

**DOI:** 10.4317/medoral.17401

**Published:** 2011-12-06

**Authors:** María Peñarrocha, David Peñarrocha, José V. Bagán, Miguel Peñarrocha

**Affiliations:** 1 Associate Professor of Oral Surgery. Valencia University Medical and Dental School; 2 Dental surgeon. Master in Oral Surgery and Implantology. Valencia University Medical and Dental School; 3 Chairman of Oral Medicine. Valencia University Medical and Dental School. Head of the Department of Stomatology, Valencia University General Hospital; 4 Chairman of Oral Surgery. Valencia University Medical and Dental School. Valencia (Spain)

## Abstract

Introduction. Trigeminal neuropathy is most often secondary to trauma. The present study explores the underlying causes and the factors that influence recovery. Material and methods. A retrospective case study was made involving 63 patients with trigeminal neuropathy of traumatologic origin, subjected to follow-up for at least 12 months. Results. Fifty-four percent of all cases were diagnosed after mandibular third molar surgery. In 37 and 19 patients the sensory defect was located in the territory innervated by the mental and lingual nerve, respectively. Pain was reported in 57% of the cases, and particularly among the older patients. Regarding patient disability, quality of life was not affected in three cases, while mild alterations were recorded in 25 subjects and severe alterations in 8. Partial or complete recovery was observed in 25 cases after 6 months, and in 32 after one year. There were few recoveries after this period of time. Recovery proved faster in the youngest patients, who moreover were the individuals with the least pain. Conclusion. Our patients with trigeminal neuropathy recovered particularly in the first 6 months and up to one year after injury. The older patients more often suffered pain associated to the sensory defect. On the other hand, their discomfort was more intense, and the patients with most pain and the poorest clinical scores also showed a comparatively poorer course.

** Key words:**Post-traumatic trigeminal neuropathy.

## Introduction

Trauma, whether accidental or iatrogenic, is the most common cause of trigeminal neuropathy (TN)(1). Most cases of post-traumatic TN are the result of oral surgical operations, particularly the removal of impacted lower third molars. The sensory defects are located mainly in the territories innervated by the inferior alveolar nerve and the lingual nerve (2) Most of these lesions are reversible, though more persistent cases can adversely affect patient quality of life (3).

The present study evaluates a series of 63 patients with post-traumatic TN, with a view to identifying the underlying causes, the clinical manifestations and the factors that influence recovery.

## Material and Methods

A retrospective case study was made of 63 patients seen for sensory alterations of the face and diagnosed with trigeminal neuropathy of traumatic origin in the period 1996-2009. The following inclusion criteria were established: patients showing a sensory defect in the mucocutaneous territory innervated by one or more trigeminal branches, produced by a traumatic process, and with a minimum follow-up period of 12 months. Those cases with a symptoms-free period between the time of trauma and the appearance of neuropathy were excluded. All patients gave informed consent to participate in the study.

No immediate anterior traumatism capable of accounting for the neuropathy was documented. We registered the presence, degree and duration of the associated discomfort, which was rated as follows: burning, itching, stabbing, flashing pain. A clinical scale was used to score the degree of disability resulting from trigeminal neuropathy: no effect (daily life activities not affected), mild (causes some concern, preventing some non-usual activity), moderate (prevents normal life, limiting certain usual activities) or severe (causes important disability).

Extraoral panoramic X-rays were obtained in all cases. Maxillofacial or cranial computed tomography scans were performed in some cases. Vitamin B complex was prescribed, and in the case of pain amitriptyline 50-75 mg/day and/or carbamazepine 400-600 mg/day was administered. The clinical course was assessed one, 6 and 12 months after the visit to the clinic, and at the last control, and was rated as full recovery, partial recovery, or no improvement.

A descriptive analysis was made of all the study variables. Comparisons between qualitative variables were made using the chi-squared test, accepting statistical significance for p<0.05.

## Results

The mean patient age was 45.4 years (range 17-76). There were 11 men and 52 women (ratio 1:5).

The following antecedents of trauma were recorded: impacted lower third molar removal (34 patients, 54%), simple extraction (9 patients), removal of impacted root fragments (4 patients), implant placement (9 patients), dental anesthesia in conservative dental treatment (4 patients), endodontic treatment (1 patient), and direct facial traumatisms (2 patients).

In 46% of the cases neuropathy was located on the left side, in 49% on the right side, and in 5% of the cases the disorder proved bilateral. The most frequently affected territory corresponded to the third trigeminal branch: the inferior alveolar nerve in 37 cases, and the lingual nerve in 19 cases. The territory of the second trigeminal branch was affected in four cases, while the second and third branches were implicated in two subjects. Lastly, all three branches of the fifth cranial nerve were affected in one case.

In addition to the sensory defect, 57% of the patients (36 cases) suffered associated pain. The pain was described as burning in 19 cases, itching in 6, stabbing in 5, and flashing or fulgurant in three. The pain was poorly defined in three patients. Regarding patient disability, quality of life was not affected in three cases, while mild alterations were recorded in 25 subjects and moderate or severe alterations in 8.

In relation to drug treatment, 5 patients received no treatment, while 25 received only vitamin B complex. Combination carbamazepine and amitriptyline was prescribed in 18 patients, while four received carbamazepine alone, and 9 received amitriptyline. Lastly, two patients received other drugs and analgesics.

After 6 and 12 months of follow-up, 25 and 32 patients showed improvement, respectively. On occasion of the last control (mean 3 years), 35 patients had improved. Full recovery took place in 9 cases within the first 6 months of follow-up, with another 7 cases over the next 6 months. After this period there were no further recoveries, however ( Table 1).

**Table 1:**
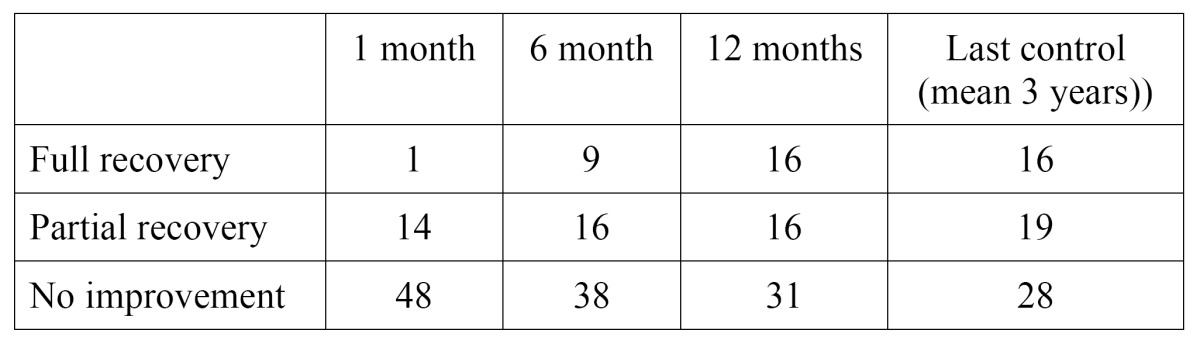
Clinical course of the patients.

Marginally significant differences were recorded on comparing the presence of pain by age intervals (chi-squared = 5.612, p=0.06). Of the patients over 60 years of age, 81.3% complained of pain. In the 41-60 years age range this proportion dropped to 55%, while the youngest patients (20-40 years of age) reported pain in 44.4% of the cases. The mean age of the patients with associated pain was 50.41 years versus 38.7 years in the case of the patients without pain (Student t-test for independent samples = -3.084, p = 0.003). A statistically significant association was observed between patient age and the presence of pain.

**Figure 1:**
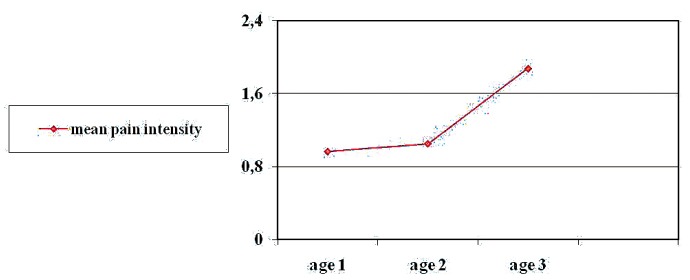
Pain intensity by age groups. Mean pain intensity, age 1, age 2, age 3.

Statistically significant differences were observed on correlating patient age to pain intensity (r = 0.385, p = 0.002). In effect, pain intensity was seen to increase with advancing age. This was corroborated by analysis of variance (ANOVA) between the age groups and pain intensity (F = 3.862, p = 0.026) – the oldest subjects reporting the greatest intensity of pain (Fig. 1).

Pain was reported by four males and 32 females, though no statistically significant differences in the presence of pain between the two sexes was observed (chi-squared = 2.350, p = 0.125), since many females were included in the study series. No correlations were found between patient sex and the characteristics, frequency or duration of pain.

**Figure 2:**
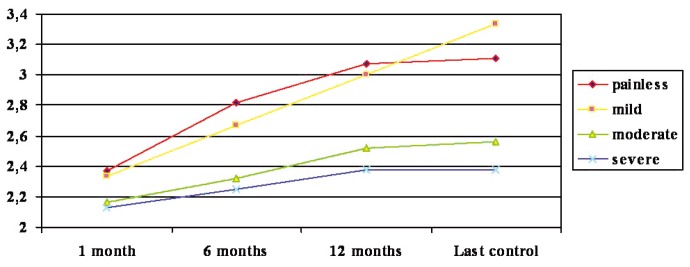
Improvement over time and initial pain. (Y: mean improvement). No pain, mild, moderate, severe, 1 month, 6 months, 12 months, last control.

In contrast, a significant relationship was observed between pain intensity and the clinical course. This relationship was examined by mixed ANOVA, taking as independent variables the time elapsed from one clinical control to the next (after 1, 6 and 12 months, and at last control) and the intensity of pain (none, mild, moderate, severe), while recovery or non-recovery was taken as the dependent variable. (Fig. 2) indicates that the patients with less intense pain showed better recovery over time.

**Figure 3:**
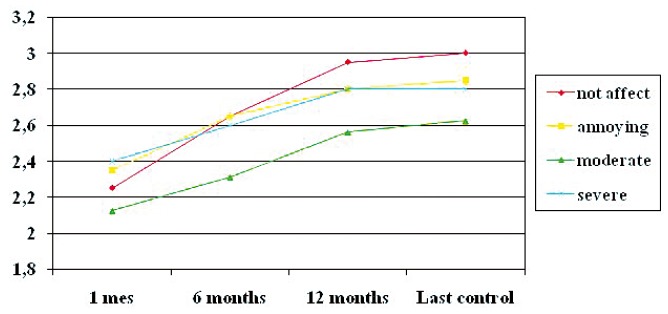
Improvement over time and clinical scale (Y: mean improvement). No effect, mild, moderate, severe, 1 month, 6 months, 12 months, last control.

Mixed, two-factor ANOVA was used to compare the clinical scale with the patient course over time, taking as first factor the time elapsed (1, 6 and 12 months, and last control), and as second factor the clinical score. Patient improvement in turn was taken as the dependent variable. The analysis revealed a statistically significant relationship, with lower clinical scores being correlated to greater mean patient improvement (Fig. 3).

## Discussion

Trigeminal neuropathy is most often secondary to trauma, with a proportion of close to 40% of all cases (1). The most common underlying cause is impacted lower third molar extraction (4,5). Fifty-four percent of our patients suffered neuropathy after the removal of lower third molars, with the observation of localized sensory defects. The territory of the inferior alveolar nerve was affected in over two-thirds of all cases, and the territory of the lingual nerve in almost one-third. Damage to the inferior alveolar nerve is explained by the anatomical proximity between the apexes of the third molar and the canal that houses the nerve (4,6). Sectioning of the crown appears to be a viable option in those cases where elimination of the entire tooth poses a significant risk of damaging the inferior alveolar nerve (7).

Likewise, due to the anatomical position of the lingual nerve in relation to the third molar, the former can be damaged during maneuvering to extract the molar (6,8-10). Schultze-Mosgau and Reich (11), in a series of 1107 molar extractions, reported neuropathy affecting the inferior alveolar nerve and lingual nerve in 2.2% and 1.4% of the cases, respectively. Fielding et al. (12), in a survey of 452 maxillofacial surgeons, found that 343 claimed to have patients with lingual sensory defects after the removal of impacted lower molars, and that these alterations proved permanent in 18.6% of the cases. Kipp et al. (13), following the extraction of 1377 lower molars, reported 60 cases of dysesthesia or hypoesthesia, and 13 of these cases were moreover permanent. Surgery of this kind poses a risk, even when maximum care is taken, and the patient should be duly informed of this fact.

Trigeminal neuropathy (TN) secondary to dental anesthesia has been little described in the literature, and was recorded in four of the cases of our series. Chan and Mulford (14) published a case of iatrogenic TN resulting from local anesthesia injection. Chin numbness after dental implant placement has been described as a consequence of direct damage of the inferior alveolar nerve caused by the surgical drill or the implant itself (15). Knowledge of the distribution of the alveolar neurovascular supply in the region of the third molar is important when performing surgical procedures that can affect these structures (16). Permanent damage of the nerve can result after inferior alveolar nerve block (17). A case has been reported after dental anesthesia in the context of surgery of this kind, with no direct damage to the nerve caused by the implant (18). The inferior alveolar nerve can be damaged during lower molar extraction as a result of direct traumatism or neurotoxicity (19), as occurred in one of our cases.

According to Karas et al. (20), sensory impairment in relation to TN is greater among women, while Sandstedt and Sörensen (4) found women to suffer more and longer lasting pain than men. Most of our patients were females, and we found no significant differences on correlating patient sex to the presence or characteristics of pain. The older patients were seen to suffer more pain. These data coincide with those published by Sandstedt and Sörensen (4). A similar situation was found in the case of postherpetic trigeminal neuralgia, where for some unknown reason the incidence was seen to increase with age – affecting 30-50% of all patients over 60 years of age (14).

The prognosis depends on the severity of the nerve damage. Kipp et al. (13), following the extraction of 1377 impacted lower third molars, reported sensory alterations in 60 cases, of which 64% resolved within the first 6 months. Of our cases, 9 showed full recovery after 6 months, while an additional 7 patients showed complete resolution after 12 months. Following this period there were practically no further recoveries, however. Some authors (21) report that the incidence of spontaneous recovery 6 months after the causal lesion is very low, and that if axonal regeneration does not take place within two years, the chances for regeneration are lost and the damage becomes permanent.

Sandstedt and Sörensen (4) examined 226 patients with trigeminal sensory disorders seeking economical compensation from insurance companies. Seventy-nine percent of the cases were a consequence of lower third molar extraction, and 70% of the patients complained of paresthesias. Women and elderly people were the subjects reporting most discomfort after nerve damage. Pain can condition the existence of both functional and psychological repercussions in these patients (22,23). Over one-half of our patients suffered pain associated to the sensory defect, particularly the older patients. On the other hand, these elderly subjects suffered more intense pain, with poorer clinical scores and a comparatively poorer course.

## References

[1] Peñarrocha MA, Mora E, Bagán JV, García B, Peñarrocha M (2009). Idiopathic trigeminal neuropathies: a presentation of 15 cases. J Oral Maxillofac Surg.

[2] Blackburn CW (1990). A method of assessment in cases of lingual nerve injury. Br J Oral Maxillofac Surg.

[3] Jerjes W, Upile T, Shah P, Nhembe F, Gudka D, Kafas P (2010). Risk factors associated with injury to the inferior alveolar and lingual nerves following third molar surgery-revisited. Oral Surg Oral Med Oral Pathol Oral Radiol Endod.

[4] Sandstedt P, Sörensen S (1995). Neurosensory disturbances of the trigeminal nerve: a long-term follow-up of traumatic injuries. J Oral Maxillofac Surg.

[5] Robert RC, Bacchetti P, Pogrel MA (2005). Frequency of trigeminal nerve injuries following third molar removal. J Oral Maxillofac Surg.

[6] Robinson PP (1992). The effect of injury on the properties of afferent fibres in the lingual nerve. Br J Oral Maxillofac Surg.

[7] Landi L, Manicone PF, Piccinelli S, Raia A, Raia R (2010). A novel surgical approach to impacted mandibular third molars to reduce the risk of paresthesia: a case series. J Oral Maxillofac Surg.

[8] Walters H (1995). Reducing lingual nerve damage in third molar surgery: a clinical audit of 1350 cases. Br Dent J.

[9] Brann CR, Brickley MR, Shepherd JP (1999). Factors influencing nerve damage during lower third molar surgery. Br Dent J.

[10] Pogrel MA, Le H (2006). Etilogy of lingual nerve injuries in the third molar region: a cadaver and histologic study. J Oral Maxillofac Surg.

[11] Schultze-Mosgau S, Reich RH (1993). Assessment of inferior alveolar and lingual nerve disturbances after dentoalveolar surgery, and of recovery of sensitivity. Int J Oral Maxillofac Surg.

[12] Fielding AF, Rachiele DP, Frazier G (1997). Lingual nerve paresthesia following third molar surgery: a retrospective clinical study. Oral Surg Oral Med Oral Pathol Oral Radiol Endod.

[13] Kipp DP, Goldstein BH, Weiss WWJr (1980). Dysesthesia after mandibular third molar surgery: a retrospective study and analysis of 1,377 surgical procedures. J Am Dent Assoc.

[14] Chang WK, Mulford GJ (2000). Iatrogenic trigeminal sensorimotor neuropathy resulting from local anesthesia: a case report. Arch Phys Med Rehabil.

[15] Bartling R, Freeman K, Kraut RA (1999). The incidence of altered sensation of the mental nerve after mandibular implant placement. J Oral Maxillofac Surg.

[16] Pogrel MA, Dorfman D, Fallah H (2009). The anatomic structure of the inferior alveolar neurovascular bundle in the third molar region. J Oral Maxillofac Surg.

[17] Pogrel MA (2007). Permanent nerve damage from inferior alveolar nerve blocks.An uptade to include articaine. J Calif Dent Assoc.

[18] Flanagan D (2002). Delayed onset of altered sensation following dental implant placement and mental block local anesthesia: a case report. Implant Dent.

[19] Pogrel MA (2007). Damage to the inferior alveolar nerve as the result of root canal theraphy. J Am Dent Assoc.

[20] Karas ND, Boyd SB, Sinn DP (1990). Recovery of neurosensory function following orthognathic surgery. J Oral Maxillofac Surg.

[21] Wofford DT, Miller RI (1987). Prospective study of dysesthesia following odontectomy of impacted mandibular third molars. J Oral Maxil¬lofac Surg.

[22] Sandstedt P, Sörensen S (1995). Neurosensory disturbances of the trigeminal nerve: a long-term follow-up of traumatic injuries. J Oral Maxillofac Surg.

[23] Peñarrocha-Diago M, Mora-Escribano E, Bagán JV, Peñarrocha-Diago M (2006). Neoplastic trigeminal neuropathy: presentation of 7 cases. Med Oral Patol Oral Cir Bucal.

